# Supplementary material for: Development of a computer-aided design software for the quantitative evaluation of aesthetic damage
Source: PLoS One. 2019 Dec 18;14(12):e0226322. doi: 10.1371/journal.pone.0226322 (PMC6919621; doi:10.1371/journal.pone.0226322)
Supplement: S1 Code site — 1. (ZIP) [file pone.0226322.s001.zip › [EESC_jr][Projeto]Relatorio_07_21/reports/templates_zip/gentelella-master/vendors/echarts/test/visualMap-opacity.html]

Color Alpha in scatter (using outOfRange as 'selected')

Opacity in bar (label color specified)

Opacity in scatter (label color specified)

Opacity in graph (label color specified)

Opacity in heatmap (inactive color default using opacity)

Opacity in pie (label color and label line color specified)
